# Understanding COVID-19 in Wuhan From the Perspective of Cold-Dampness: Clinical Evidences and Mechanisms

**DOI:** 10.3389/fmed.2021.617659

**Published:** 2021-02-22

**Authors:** Yujiao Zheng, De Jin, Jiaran Lin, Yuehong Zhang, Jiaxing Tian, Fengmei Lian, Xiaolin Tong

**Affiliations:** ^1^Department of Endocrinology, Guang'anmen Hospital, China Academy of Chinese Medical Sciences, Beijing, China; ^2^Graduate School, Beijing University of Chinese Medicine, Beijing, China

**Keywords:** COVID-19, cold-dampness, cold-dampness plague theory, traditional Chinese medicine, infectious disease

## Abstract

Traditional Chinese medicine (TCM) has played a significant role in the treatment of coronavirus disease 2019 (COVID-19) in Wuhan City. During the epidemic, Academician Tong Xiaolin suggested a close association of COVID-19 with cold-dampness, an etiological factor in TCM, by summarizing the characteristics of the COVID-19 patients in Wuhan. and the theory of Cold-dampness Plague was proposed. Based on the Cold-dampness Plague theory, a series of TCM drugs, such as Huoxiang Zhengqi Dropping Pills, Lianhua Qingwen Granules Hanshiyi Formula, and Tongzhi Granule were developed for the different stages, namely mild, moderate, severe, recovery, of the COVID-19. In addition, clinical evidences were obtained through randomized clinical trials or retrospective cohort studies. The Anti-SARS-CoV-2 mechanism of the TCM prescriptions were then summarized from the four aspects: targeting the ACE2 and 3CLPro, targeting cytokines, targeting acute immune responses to SARS-CoV-2, and targeting pulmonary fibrosis. Despite the clinical efficacy and therapeutic pharmacology speculation, more studies such as large-scale randomized clinical trials, cell and animal experiments are needed to further verify the theory of the Cold-dampness Plague in COVID-19 patients.

## Introduction

In December 2019, coronavirus disease 2019 (COVID-19), which first broke out in Wuhan City, the capital of Hubei province in China, was identified as being caused by a novel coronavirus named severe acute respiratory syndrome coronavirus 2 (SARS-CoV-2) (1, 2). The disease was declared a pandemic by the World Health Organization (WHO), when it rapidly spread across China and rapidly reached other Asian regions, the USA, and some European countries (3–5). Fever, cough, myalgia, or fatigue were the common clinical manifestations in COVID-19 patients, dyspnea usually occurred in severe cases, and was life-threatening (6). Up to 19 September, 2020, 30,369,778 individuals were infected by COVID-19 globally, including 948,795 death cases (7).

It is worthy of note that in this battle, traditional Chinese medicine (TCM) played a significant role in the prevention, treatment and rehabilitation of COVID-19 in China (8). TCM therapy plan was an indispensable part of the Diagnosis and Treatment Guideline for COVID-19 (3rd−8th edition) released by the National Health Commission of the People's Republic of China (9). According to the statistics, as of March 6, 2020, TCM therapy was being administered in 92.36% of COVID-19 patients in China (10). In contrast to Western medicine, TCM, as one of the primary alternative and complementary medicine, has its own unique theoretical system and methods of understanding human physiology and pathology. Over thousands of years of its clinical use, TCM has accumulated rich experience in the face of more than 300 documented plagues in Chinese history (11).

Considering the symptom observed in COVID-19 patients and environmental and climatic conditions in Wuhan during the epidemic, Academician Tong Xiaolin suggested that the epidemic was closely associated with the pathological stage of cold-dampness, an etiological factor in TCM. Based on the theory of cold-dampness, a series of Chinese medical prescriptions and treatment plans have been developed for COVID-19 patients (Table 1). At the same time, there have been reports that TCM therapy can strengthen immunity, exert anti-inflammatory activity, and promote the elimination of virus from the body, to achieve recovery and prevent complications in COVID-19 patients (16, 17). In this review, to fully understand the role of TCM in the prevention and treatment of the epidemic, from the perspective of cold-dampness, we summarize the research regarding its theory, treatments, clinical evidence, and mechanisms for COVID-19 in Wuhan.

**Table 1:** Summary of the included studies.

**Study ID**	**Sample size**	**Intervention method**	**Duration of interventions**	**Complications**	**Primary outcome**
Xiao et al. (12)	*n* = 182 (LQG group *n* = 58, LQG+HZDP group *n* = 61, WM group *n* = 63) Male: LQG group 60.3%, LQG+HZDP group 54.1%, WM group 55.6% Mean age (SD): LQG group 52.86 ± 13.95, LQG+HZDP group 56.07 ± 12.10, WM group 53.90 ± 13.92	RCTHZDP (Pogostemon cablin (Blanco) Benth, Atractylodes lancea (Thunb.) DC., Magnolia officinalis Cortex, Angelicae dahurica Radix, Poria cocos (Schw.) Wolf, Areca catechu L., Pinellia ternate (Thunb.) Breit., Glycyrrhizae Radix et Rhizoma, Perilla frutescens, and Citrus reticulata), 2.6 g, twice a day.LQG (Forsythia suspensa (Thunb.) Vahl, Ephedra sinica Stapf, Lonicera japonica Thunb., Isatis indigotica Fortune, Mentha haplocalyx Briq., Dryopteris crassirhizoma Nakai, Rhodiola rosea L., Gypsum Fibrosum, Pogostemon cablin (Blanco) Benth., Rheum palmatum L., Houttuynia cordata Thunb., and Glycyrrhiza uralensis Fisch. Armeniaca sibirica (L.) Lam), 6 g, three times a day.	LQG group: 12.47 ± 3.16 days LQG+HZDP group: 12.79 ± 2.94 days WM group: 13.14 ± 2.54 days	Bronchial asthma, chronic obstructive pulmonary disease, coronary artery disease, high blood pressure, diabetes, hyperlipidemia, and others.	The proportion of patients who progressed to severe status and clinical symptoms
Tian et al. (13)	n = 721 (treatment arm *n* = 430, control arm *n* = 291) Male: 48.1% Mean age (SD): 48.49 ± 14.362 *n* = 721 (treatment arm *n* = 430, control arm *n* = 291) Male: 48.1% Mean age (SD): 48.49 ± 14.362	Retrospective cohort studyHSYF (Ephedrae Herba, Gypsum fibrosum, Armeniacae Semen, Notopterygii Rhizoma seu Radix, Lepidii/Descurainiae Semen, Cyrtomii Rhizoma, Pheretima, Cynanchi paniculati Radix, (Pogostemonis Herba, Eupatorii Herba, Atractylodis Rhizoma, Poria, Atractylodis macrocephalae Rhizoma, Crataegi Fructus, Massa medicate fermentata, Hordei Fructus germinates, Magnoliae officinalis Cortex, Arecae Semen, Tsaoko Fructus and Zingiberis Rhizoma recens) including decoction, granules, etc.	At least 2 days. If there is no adverse effect or disease progression, HSYF can be taken continuously until recovery.	Hypertension (16.9%), coronary heart disease (4.6%), diabetes (7.1%), bronchial asthma (3.3%), chronic obstructive pulmonary disease (1.2%), hyperlipidemia (6.7%), fatty liver (7.9%), gallbladder disease (2.4%), thyroid disease (2.1%), stroke (0.3%), chronic glomerulonephritis (0.8%), cancer (0.8%), hepatitis (2.1%), tuberculosis (0.4%), and other diseases (14.3%).	The proportion of mild and moderate COVID-19 patients who progressed to a severe disease status
Chen et al. (14)	*n* = 662 (treatment arm *n* = 484, control arm *n* = 178) Male: 44.7% Mean age (SD):60 (47–70)	Retrospective cohort studyHXF (Poria, Astragali Radix, Pogostemon cablin (Blanco) Benth., Prunus armeniaca L. var. ansu Maxim., Pinellia ternata (Thunb.) Breit., Ephedra sinica Stapf, Cinnamomum cassia Presl, Eupatorium fortunei Turcz, Codonopsis Radix), 200 mL each time, twice a day in hospital days.	NR	Chronic obstructive lung disease (2.9%), hypertension (31.4%), cardiovascular disease (8.0%), diabetes (14.2%), malignancy (1.8%), cerebrovascular disease (5.7%), chronic kidney disease (0.6%), and chronic liver disease (1.2%).	Mortality rate
He et al. (15)	*n* = 420 (treatment arm *n* = 325, control arm *n* = 95) Male:49.0% Mean age (SD): 56 (43–63.75)	Retrospective observational StudyTZG (Astragali Radix 15 g, Codonopsis Radix 15 g, Atractylodis Macrocephalae Rhizoma 15 g, Adenophorae Radix 15 g, Glehniae Radix 15 g, Ophiopogonis Radix 15 g, Citri Reticulatae Pericarpium 15 g, Poria 15 g, Pinelliae Rhizoma Praeparatum 9 g, Anemarrhenae Rhizoma 12 g, Salviae Miltiorrhizae Radix et Rhizoma 15 g, Fritillariae Thunbergii Bulbus 15 g, Paeoniae Radix Rubra 15 g, Platycodonis Radix 15 g, Saposhnikoviae Radix 9 g, Glycyrrhizae Radix et Rhizoma 6 g, Fructus Hordei Germinatus 9 g, Crataegi Fructus 9 g, Massa Medicata Fermentata 9 g, Rhizoma Dioscoreae 15 g), administered to 1 bag (dissolved in 200 mL of water at 95°C) per day, twice a day.	Median course of disease was 40 days	Hypertension (26.4%), hyperlipidemia (10.7%), diabetes (10.5%), coronary heart disease (5.5%), hepatopathy (3.1%), chronic bronchitis (2.9%), hyperuricemia malignant tumor (1.7%), chronic nephritis (1.0%), and cerebral apoplexy (1.0%).	RT-PCR test result of the observed subjects at the end of quarantine

## TCM Theory of COVID-19 From the Perspective of Cold-Dampness

Based on TCM theory, Tong proposed the role of cold-dampness in the pathogenesis of COVID-19. Unlike Western medicine, TCM often considers the pathogenesis of the disease from a holistic perspective, which includes the disease itself, the environment state without, and the body state within. There is accumulating evidence, from all three aspects, that supports the theory of cold-dampness in COVID-19. Etiological studies have shown that an increase in temperature can accelerate the inactivation of SARS-CoV-2. An increment in temperature, from 24 to 35°C, resulted in faster virus decay and shorter half-life at relative humidity (18, 19). An increase in atmospheric temperature dampened the transmission of the virus, whereas a decrease resulted in new cases of COVID-19; a peak, in the growth of SARS-CoV-2, was observed in the cold season (20). In addition, isolation of SARS-CoV-2 on conditioned frozen food has been suggested as indication of its role as possible source of COVID-19 in one report (21). Under refrigerated (at 4°C) and freezing conditions (from−10°C to −80°C), SARS-CoV-2 remained highly stable on frozen food for 14–21 days, thereby indicating the cold nature of the epidemic (22). Although some researchers are of the opinion that the relative humidity is negatively related to the spread of COVID-19 (23), based on the evidence regarding environmental factors and human body state, we believe that the epidemic has damp characteristics.

As for the environmental state, according to local meteorological data, the precipitation in Wuhan, in January 2020, was 4.6 times the average precipitation observed in the same period during the last 20 years. In a study, that investigated the meteorological factors of the COVID-19, 1% increase in precipitation led to 0.07% of decline in recovery cases and 1 and 0.86% increase in confirmed cases and deaths, respectively (24). In addition, an increase in humidity in the air, during the winter season, can reduce host innate immune response and enhance microbial growth in a closed environment, which can easily worsen the underlying health conditions (especially respiratory illnesses) (25).

According to the theory of Cold-dampness Plague, the COVID-19 comprises of following four stages: (a) mild status in the early stage, (b) moderate status in the middle stage, (c) severe and critical status in the late stage, and (d) rehabilitation status in the recovery stage (26). In the early stage of the disease, usually no manifestations of pneumonia are present on medical imaging, and the cold-dampness constraint in the lung is the major pathological factor. In this stage, the patients present with no or mild clinical symptoms such as fever, aversion to cold, fatigue, sometimes accompanied by muscular weakness, anorexia, diarrhea, etc. In the middle stage of the disease, imaging findings are suggestive of pneumonia, and the cold-dampness obstructing the lung is the primary pathological factor, and patients usually manifest fever, cough, asthma, abdominal distension, and constipation. In the late stage, due to the cold-dampness pathogen attacking the internal viscera and a general deficiency of *qi*, acute respiratory distress, including respiratory failure, accompanied with other organ failure appears; an obvious progression of >50% in the lesion, within 24–48 h, is observed on chest imagings (14). The symptoms of dyspnea, coma, dysphoria, sweating and cold limbs usually manifest in severe and critical cases. In the recovery stage, although consecutively negative RT-PCR tests and improvement in chest radiography are obtained, the patients usually have mild clinical symptoms such as shortness of breath, fatigue and poor appetite. This phenonmenon is mainly due to remaining cold-dampness pathogen in the body (27). Despite the fact that symptoms at different stages are varied, the cold-damp plague attacking the respiratory system and subsequently involving other organs, as the diasease progresses, is the core pathogenesis of COVID-19. According to TCM theory, the treatment is aimed to dispel cold and remove dampness, thereby regulating the whole body state, promoting the excretion of the virus, and restoring the immunity.

## The Clinical Evidences of Cold-Damp Nature of COVID-19

### Treatment of Suspected and Diagnosed Cases of COVID-19 (12)

For suspected and mild cases of COVID-19, TCM prescriptions, Huoxiang Zhengqi Dropping Pills (HZDP) and Lianhua Qingwen Granules (LQG) were prescribed from the perspective of cold-dampness nature in the early and middle stage of the cold-damp plague. Under the guideline of the cold-dampness TCM theory, HZDP was administered for the pathogenesis of dampness and LQG for the cold; thus, the combination of HZDP and LQG was recommended to dispel cold-dampness pathogens from the body. Both two TCM prescriptions have been widely applied in the infectious diseases such as Severe Acute Respiratory Syndrome (SARS) and influenza in China before (28–30). In our randomized controlled trial (RCT) of HZDP and LQG, conducted in Wuhan, 188 diagnosed and 95 suspected COVID-19 patients were enrolled. The patients were randomly divided into three group, namely the LQG, a combination of HZDP and LQG and western medicine group, in a ratio of 1:1:1. Among the three groups, the utilization rate of anti-infective drugs (including oseltamivir, arbidol and macrolide antibiotics) was significantly higher in the western medicine group (*P* < 0.05). After 14 days of treatment, among the 182 diagnosed COVID-19 patients who completed the study, the proportion of patients who progressed to severe status was lowest in the HZDP+LQG group (1.6%), when compared to the LQG group (8.6%) and the western medicine group (11.1%). With respect to the symptom improvement, in all three intervention groups, fever and diarrhea was alleviated, whereas the HZDP+LQG group had obvious advantages in relieving nausea, vomiting, and limb soreness. Above all, the results of our study suggested that the combination of HZDP and LQG developed from the cold-dampness perspective has potential advantages in the treatment of suspected and diagnosed cases of COVID-19.

### Treatment of Mild and Moderate Cases of COVID-19 (13)

Under the guidance of cold-damp plague theory, a TCM prescription. Hanshiyi Formula (HSYF), was formulated for mild and moderate COVID-19 patients; HSYF was composed of herbs meant to reduce lung inflammation and expel cold-dampness from the body. In our cohort study, 721 patients with mild and moderate COVID-19, from 17 quarantine stations in Wuchang District of Wuhan, were enrolled. Of total, 430 patients received HSYF (exposed group) and 291 did not receive HSYF (control group). In the exposed group, none of the patients (0.0%) turned severe; however, 19 patients (6.5%, *P* < 0.001) in the control group transitioned to severe status. The difference between the two groups in terms of progression to severe disease (exposed group-control group) was −6.5 % [95% CI: (−8.87%, −4.13%)]. Considering the difference in sample size between the two groups and the imbalance of confounding factors, a univariate logistic regression analysis was used. After a 1:1 ratio of propensity score matching (PSM), the sample size of HSYF users and non-users were both 148, and it's found that no HSYF users progressed to severe status of COVID-19, whereas 4.7% non-users turned to severe status, the difference between the two groups was −4.7 % [95% CI: (−8.2%, −1.2%)]. Comparing with the 14% of cases which can develop into severe status according to the report of the WHO-China joint mission (31), the result of the study showed that HSYF was effective in reducing the progression of mild and moderate COVID-19 patients to severe status. However, further larger scale of clinical studies are required to further verify the result.

### Treatment on the Severe and Critical Cases of COVID-19 (14)

According to the TCM theory of COVID-19 from the cold-dampness perspective, severe and critical COVID-19 patients developed respiratory system failure, often accompanied with other organs failure resulting from the cold-dampness pathogen in the respiratory tract attacking the internal viscera. Based on the TCM theory and the disease prognosis, Hexin Formula (HXF) was developed to halt progression of the disease and to treat the severe and critical COVID-19 patients. Although currently there is no appropriate treatment plan for severe and critical COVID-19 patients, there is evidence supporting TCM, as besides providing supportive treatment, it can aid in the treatment of severe COVID-19 (32). In our retrospective cohort study of 662 patients in Wuhan with severe and critical COVID-19, the mortality risk of TCM users was reduced by 82.2% (odds ratio 0.178, 95% CI 0.076–0.418; *P* < 0.001), when compared to the non-users, suggesting that HXF guided by the TCM theory of cold-damp plague may reduce the mortality and can be used as an alternative treatment option besides conventional antiviral and supportive treatment.

### Treatment of COVID-19 Patients in the Recovery Stage With Positive RT-PCR Test Results (15)

Among recovered COVID-19 patients, some have recurrent transcriptase–polymerase chain reaction (RT-PCR) test results. This phenomenon of recurrent positive RT-PCR test results further adds to the difficulty in controlling COVID-19, not only in China but also around the world. At present, there is no specific treatment for these individuals, as they usually manifest as no or mild symptoms, and their infectivity is also uncertain. According to TCM theory, this is mainly because of lack of healthy visceral *qi*, and remaining cold-dampness pathogen in the body. Considering the role of cold-damp plague in the recovery stage, a universal TCM prescription, Tongzhi Granule (TZG), was developed, that focused on nourishing healthy *qi* and expelling residual cold-damp pathogen from the body. In our retrospective cohort study of 420 recovered COVID-19 patients with positive RT-PCR results, the recurrence rate of positive RT-PCR test results was lower in the TZG group, when compared to a control group (2.8% [9/325] vs. 15.8% [15/95]). Thus, indicating that TCM intervention using TZG guided by the cold-damp plague theory may play a positive role in reducing the RT-PCR test results in the patients recovered from COVID-19.

## Anti-SARS-CoV-2 Mechanism and Therapeutic Pharmacology

SARS-CoV-2 (2019-nCoV) is a member of the family coronaviridae and genus betacoronavirus, and is closely related to two bat-derived SARS-like coronaviruses (bat-SL-CoVZC45 and bat-SL-CoVZC21) (33). Although CoVs have species diversity, they share key genomic elements. Sequence analysis showed that the 2019-nCoV has structural features typical of coronavirus genome (34). Typical CoV genome and subgenome contain six open reading frames (ORFs), which encode 16 non-structural proteins (NSP 1-16), except for γ coronavirus, which lacks NSP 1. There is a −1 frameshift between ORF1a and ORF1b; 5' ORF1 a/b encodes the polypeptides pp1a and pp1ab. These polypeptides are processed into 16 NSPs by virus-encoded enzymes, such as 3C-like protease (3CLPro), master protease (mPro), and one or two papain like protease (PLpro) (35, 36).

RNA-dependent RNA polymerase RdRp and ExoN enzymes, involved in virus transcription and replication (37), are potential broad-spectrum anti-CoV targets (38). Angiotensin-converting enzyme two (ACE2) is a functional receptor for SARS-Co-2. Therefore, much of the research on the anti-CoV mechanism is focused on 3CLPro, PLpro, ACE2, RdRp and proofreading ExoN. In addition, the cytokine storms and acute immune responses are also important targets. It is widely known that the underlying mechanism in herbal medicines are “multi-component, multi-target, and multi-pathway” (39–41). Traditional Chinese medicine (TCM) plays a vital role and provides unique advantages in the management of COVID-19. The possible anti-SARS-CoV-2 mechanisms of the TCM prescriptions, including HZDP, LQG, HSYF, HXF and TZG are shown in Tables 2–6, respectively.

**Table 2:** Herbal medicine of HZDP used in the treatment of COVID-19 according to their effects, targets and mechanisms of action.

**Herbal formula**	**Herbal medicine (components)**	**Effects**	**Targets**	**Mechanism of action**	**Reference**
HZDP	Pogostemon cablin (Blanco) Benth (Patchouli alcohol)	Anti-H1N1 Influenza Virus	RLH pathway	Inhibited the expression of cytokines and the mRNA of RLH pathway.	(42)
	Atractylodes lancea (Thunb.) DC. (Atractylodes lactone Iand III)	Anti-inflammatory activity	TNF-α, IL-1β and IL-6	Promoted the expression of cytokines in inflammatory macrophages.	(43)
	Magnolia officinalis Cortex (Honokiol)	Resisted lung injury	TLR4-NF-κB pathway, Th17/Treg cells	Inhibited TLR4-NF-κB pathway-mediated inflammatory response or regulated the balance of Th17/Treg cells.	(44)
	Pinellia ternate (Thunb.) Breit. (Alkaloid)	Had protective effect on pulmonary epithelial cells	NO, TNF-α, IL-8 and ICAM-1	Inhibited the release of NO, TNF-α. Inhibited the expression of IL-8 and ICAM-1.	(45)
	Perilla frutescens (P. frutescens extract)	Regulated the inflammatory activities	SFKs (Src and Lyn) and mobilization of intracellular Ca2+	Inhibited fMLF-induced phosphorylation of the Src family kinases (SFKs), Src (Tyr416) and Lyn (Tyr396). Reduced their enzymatic activities.Decreased intracellular Ca2+ levels ([Ca2+] i).	(46)
	Angelicae dahurica Radix	Regulated the inflammatory activities	IL-1β, IL-6, IL-8, IFN-γ, NF-κB, COX-2 and iNOS	Reduced the expressions of IL-1β, IL-6, IL-8, IFN-γ, NF-κB, COX-2 and iNOS protein levels.	(47)
	Citrus reticulata (Citrus reticulata essential oil)	Had preventive effects on pulmonary fibrosis in rats	CTGFprotein, mRNA, Collagendeposition.	Adjusted the unbalance of oxidation and antioxidation.Down-regulated CTGF protein and mRNA expressions.Reduced collagendeposition.	(48)
	Poria cocos (Schw.) Wolf (Polysaccharide)	Enhanced humoral and cellular immunity	Splenocytes,IL-12p70 and TNF-α	Improved proliferation of splenocytes. Stimulated IL-12p70 and TNF-α productions in dendritic cells and macrophages.	(49)
	Platycodonis Radix (Total Saponins)	Improved inflammatory reactions	IRG-1, IL-6, IL-1β, TNF-α and ROS	Inhibited the expression of IRG-1. Reduced contents of IL-6, IL-1β, TNF-α and ROS.	(50)
	Glycyrrhizae Radix et Rhizoma (Glycyrrhizin)	reduced the severity of an infection with COVID-19	ACE2	Reduced the expression of ACE2 in theLung.	(51, 52)
	Jujubae Fructus (Polysaccharides)	Had anti-inflammatory activity	IL-6, TNF-α	Suppressed proinflammatory cytokines, such as IL-6 and TNF-α.	(53)
	Areca catechu L. (Extract of Areca catechu)	Had anti-inflammatory activity	None	None	(54)
	Zingiberis Rhizoma Recens (Aqueous extract)	Had anti-inflammatory activity	macrophage, neutrophils, monocyte and leukocyte	Inhibited macrophage and neutrophils activation as well as negatively affected monocyte and leukocyte migration.	(55)

**Table 3:** Herbal medicine of LQG used in the treatment of COVID-19 according to their effects, targets and mechanisms of action.

**Herbal formula**	**Herbal medicine (components)**	**Effects**	**Targets**	**Mechanism of action**	**Reference**
LQG	Forsythia suspensa (Thunb.) Vahl (Forsythoside A)	Controlled influenza A virus (IAV) infection and improved the prognosis of IAV infection	TLR7, MyD88, TRAF6, IRAK4 and NF-κB p65 mRNA	Inhibited influenza A virus replication.	(56)
	Lonicera japonica Thunb. (Chlorogenic acid)	Inhibited influenza A (H1N1/H3N2) virus	NP protein	Downregulated the NP protein expression, acted as a neuraminidase blocker.	(57)
	Ephedra sinica Stapf [(+)-catechin]	Inhibited the growth of influenza A PR8 virus	endosomes and lysosomes	Inhibited the acidification of intracellular compartments such as endosomes and lysosomes.	(58)
	Armeniacae Semen Amarum (amygdalin)	Slowed the progression of pulmonary fibrosis	collagen I (Col1), collagen III (Col3)	Inhibited the expression of collagen I (Col1), collagen III (Col3).	(59)
	Gypsum Fibrosum	Attenuates heat-induced hypothalamic inflammation	interleukin (IL)-1β	Inhibited heat-induced proinflammatory factors.	(60)
	Isatis indigotica Fortune (Erucic acid)	Exhibited broad-spectrum antiviral activity against influenza A virus (IAV)	NF-κB and p38 MAPK signaling	Suppressed activation of p38 MAPK and NF-κB signaling.	(61)
	Cyrtomii Rhizoma 4-hydroxybenzylideneacetone and (HBAc) 3, 4-dihydroxybenzylideneacetone (DHBAc)	Exerted anti-inflammatory effects	IκB and c-JUN pathways	Decreased the secretion of interleukin-1β.	(62)
	Houttuynia cordata Thunb. (Houttuynia cordata polysaccharides)	Have preventive effects on acute lung injury	leukocytes, serum complement	Reduced pulmonary edema, protein exudation, the deposit of complement beginning products. Reduced the number of leukocytes and restored serum complement levels.	(63)
	Pogostemon cablin (Blanco) Benth. (Patchouli alcohol)	Anti-H1N1 Influenza Virus	RLH pathway	Inhibited the expression of cytokines and the mRNA of RLH pathway.	(42)
	Rheum palmatum L. (Rhein)	Inhibited influenza A virus (IAV)	TLR4, Akt, p38, JNK MAPK, and NF-κB signal pathways	Suppressed IAV-induced oxidative stress and activated TLR4, Akt, p38, JNK MAPK, and NF-κB signal pathways.	(64)
	Rhodiola rosea L. (salidroside)	Has immunomodulatory effects	ROS,NO	Reduced the production of ROS and promoted the production of NO in activated peritoneal macrophages.	(65)
	Mentha haplocalyx Briq. (peppermint oil)	Inhibited herpes simplex virus type 1 (HSV-1) and herpes simplex virus type 2 (HSV-2)	None	Blocked virus adsorption.	(66)
	Glycyrrhiza uralensis Fisch. Armeniaca sibirica (L.) Lam (Glycyrrhizin)	May reduce the severity of an infection with COVID-19	ACE2	Reduced the expression of ACE2 in the lung.	(51, 52)

**Table 4:** Herbal medicine of HSYF used in the treatment of COVID-19 according to their effects, targets and mechanisms of action.

**Herbal formula**	**Herbal medicine (components)**	**Effects**	**Targets**	**Mechanism of action**	**Reference**
HSYF	Ephedrae Herba [(+)-catechin]	Inhibited the growth of influenza A PR8 virus	endosomes, lysosomes	IInhibited the acidification of intracellular compartments such as endosomes and lysosomes.	(58)
	Gypsum fibrosum	Attenuates heat-induced hypothalamic inflammation	interleukin (IL)-1β	Inhibited heat-induced proinflammatory factors.	(60)
	Armeniacae Semen (amygdalin)	Slowed the progression of pulmonary fibrosis	collagen I (Col1), collagen III (Col3)	Inhibited the expression of collagen I (Col1), collagen III (Col3).	(59)
	Notopterygii Rhizoma seu Radix (extract of notopterygium)	Inhibited the development of asthma	Th1 / Th2 cells, p38 signaling pathway	Changed Th1 / Th2 cells balance.Inhibited p38 signaling pathway.	(67)
	Lepidii/Descurainiae Semen	Alleviated eosinophilic inflammation	Th2 cell	Inhibited T helper 2 (Th2) cell differentiation.	(68)
	Cyrtomii Rhizoma (4-hydroxybenzylideneacetone and (HBAc) 3, 4-dihydroxybenzylideneacetone (DHBAc))	Exerted anti-inflammatory effects	IκB and c-JUN pathways	Decreased the secretion of interleukin-1β.	(62)
	Pheretima (Pheretima aspergillum decoction)	Suppressed inflammation and relieved asthma	IL-4, IL-5, IL-13, IgE, NF-κB signaling	Decreased the mRNA and protein levels of IL-4, IL-5 and IL-13 and downregulated IgE.Inhibited the activation of NF-κB signaling.	(69)
	Cynanchi paniculati Radix (Cynanchum paniculatum (Bge.) Kitag extract)	Suppressed bovine viral diarrhea (BVD) virus			(70)
	Pogostemonis Herba (Patchouli alcohol)	Anti-H1N1 Influenza Virus	RLH pathway	Inhibited the expression of cytokines and the mRNA of RLH pathway.	(42)
	Eupatorii Herba (flavonoids)	Have antibacterial activities	Staphylo tetragenus, staphyloccocus aureus, Escherichia coli and bacillus subtilis	None	(71)
	Atractylodis Rhizoma (Volatile oil)	Has anti-inflammatory effect	PGE2	Inhibited prostaglandin (PGE2) generation in the relevant tissue.	(72)
	Poria (polysaccharide)	Enhanced humoral and cellular immunity	Splenocytes,IL-12p70 and TNF-α	Improved proliferation of splenocytes. Stimulated IL-12p70 and TNF-α productions in dendritic cells and macrophages	(49)
	Atractylodis macrocephalae Rhizoma (Atractylodes lactoneIand III)	Anti-inflammatory activity	TNF-α, IL-1β and IL-6	Promoted the expression of cytokines in inflammatory macrophages.	(43)
	Crataegi Fructus (Maslinic acid)	Had anti-inflammatory properties	hGIIA-sPLA2-induced THP-1 cell	Inhibited hGIIA-sPLA2-induced THP-1 cell differentiation and migration.Binded and inhibited hGIIA-sPLA2 enzymatic activity.	(73)
	Massa medicate fermentata	None	None	None	None
	Hordei Fructus germinates (Glutamine-rich protein, hemicellulose-rich fiber)	Reduce the epithelial inflammatory response	STAT3, NFkB	Depressed signal transducer and activator of transcription 3 (STAT3) expression and inhibited nuclear factor kappa B (NFkB) binding activity.	(74)
	Magnoliae officinalis Cortex (Honokiol)	Resisted lung injury	TLR4-NF-κB pathway, Th17/Treg cells	Inhibited TLR4-NF-κB pathway-mediated inflammatory. response or regulated the balance of Th17/Treg cells.	(44)
	Arecae Semen (crude extract of Areca catechu)	Repressed the PGE 2 and arachidonic acid-induced inflammation	None	None	(54)
	Zingiberis Rhizoma recens (Aqueous extract)	Had anti-inflammatory activity	macrophage, neutrophils, monocyte and leukocyte	Inhibited macrophage and neutrophils activation as well as negatively affected monocyte and leukocyte migration.	(55)
	Tsaoko Fructus (Methanolic extract)	Had analgesic and anti-inflammatory properties.	nitric oxide	Attenuated nitric oxide production in lipopolysaccharide simulated BV2 microglia.	(75)

**Table 5:** Herbal medicine of HXF prescriptions used in the treatment of COVID-19 according to their effects, targets and mechanisms of action.

**Herbal formula**	**Herbal medicine (components)**	**Effects**	**Targets Gene**	**Mechanism of action**	**Reference**
HXF	Ephedra sinica Stapf [(+)-catechin]	Inhibited the growth of influenza A PR8 virus	endosomes and lysosomes	Inhibited the acidification of intracellular compartments such as endosomes and lysosomes.	(58)
	Cinnamomum cassia Presl (Volatile oil, cinnamaldehyde)	Had anti-influenza virus activities in the cellular level	TLR7 signaling pathway, IRAK-4, IFN-β	Activated TLR7 signaling pathway and interleukin-1 related acceptor kinase-4 (IRAK-4).Increased the expression of interferon-β (IFN-β).	(76)
	Prunus armeniaca L. var. ansu Maxim. (Amygdalin)	Slowed the progression of pulmonary fibrosis	collagen I (Col1), collagen III (Col3)	Inhibited the expression of collagen I (Col1), collagen III (Col3)	(59)
	Poria cocos (Schw.) Wolf. (Polysaccharide)	Enhanced humoral and cellular immunity	Splenocytes,IL-12p70 and TNF-α	Improved proliferation of splenocytes. Stimulated IL-12p70 and TNF-α productions in dendritic cells and macrophages.	(49)
	Pinellia ternata (Thunb.) Breit. (Alkaloid)	Had protective effect on pulmonary epithelial cells	NO,TNF-α,IL-8 and ICAM-1	Inhibited the release of NO, tumor necrosis factor-α (TNF-α). Inhibited the expression of interleukin 8 (IL-8) and intercellular cell adhesion molecule-1 (ICAM-1).	(45)
	Pogostemon cablin (Blanco) Benth. (Patchouli alcohol)	Anti-H1N1 Influenza Virus	RLH pathway	Inhibited the expression of cytokines and the mRNA of RLH pathway.	(42)
	Eupatorium fortunei Turcz. (flavonoids)	Have antibacterial activities	Staphylo tetragenus, staphyloccocus aureus,escherichia coli and bacillus subtilis	None	(71)
	Astragali Radix (astragalus polysaccharide)	Attenuated the immune stress	AMPK/SIRT-1 signaling pathway	Activated the AMPK/SIRT-1 signaling pathway.	(77)
	Codonopsis Radix (Codonopsis pilosula polysaccharides)	Ameliorated the inflammatory response	IL-6, IL-8, and TNF-α	Decreased levels of interleukin (IL)-6, IL-8, and tumor necrosis factor (TNF)-α.	(78)

**Table 6:** Herbal medicine of TZG used in the treatment of COVID-19 according to their effects, targets and mechanisms of action.

**Herbal formula**	**Herbal medicine (components)**	**Effects**	**Targets Gene**	**Mechanism of action**	**Reference**
TZG	Astragali Radix (Astragalus polysaccharide)	Attenuated the immune stress	AMPK/SIRT-1 signaling pathway	Activated the AMPK/SIRT-1 signaling pathway.	(77)
	Codonopsis Radix (Codonopsis pilosula polysaccharides)	Ameliorated the inflammatory response	IL-6, IL-8, and TNF-α	Decreased levels of interleukin (IL)-6, IL-8, and tumor necrosis factor (TNF)-α.	(78)
	Atractylodis Macrocephalae Rhizoma (Atractylodes lactone Iand III)	Anti-inflammatory activity	TNF-α, IL-1β and IL-6	Promoted the expression of cytokines in inflammatory macrophages.	(43)
	Adenophorae Radix	Enhanced immunological function	mononuclear macrophages	Enhanced the phagocytic function of mononuclear macrophages.	(79)
	Glehniae Radix (Imperatorin)	Had anti-inflammatory effects	iNOS and COX-2	Inhibited elevated iNOS and cyclooxygenase-2 (COX-2) protein expression.	(80)
	Ophiopogonis Radix (Saponin)	Regulated the function of the immune system	Macrophage, nitric oxide (NO), interleukin-1(IL-1)	Exhibited macrophages-modulating activity.	(81)
	Citri Reticulatae Pericarpium (Citrus reticulata essential oil)	Had preventive effects on pulmonary fibrosis in rats	CTGFprotein, mRNA, Collagendeposition.	Adjusted the unbalance of oxidation and antioxidation.Down-regulated CTGF protein and mRNA expressions.Reduced collagendeposition.	(48)
	Poria (Polysaccharide)	Enhanced humoral and cellular immunity	Splenocytes,IL-12p70 and TNF-α	Improved proliferation of splenocytes. Stimulated IL-12p70and TNF-α productions in dendritic cells and macrophages.	(49)
	Pinelliae Rhizoma Praeparatum (Alkaloid)	Have protective effect on pulmonary epithelial cells	NO,TNF-α,IL-8 and ICAM-1	Inhibited the release of NO, tumor necrosis factor-α (TNF-α). Inhibited the expression of interleukin 8 (IL-8) and intercellular cell adhesion molecule-1 (ICAM-1).	(45)
	Anemarrhenae Rhizoma (Timosaponin B-II)	Inhibited inflammatory responses	IL-1 β, TNF – α, IL-6, NF-kappaB	Reduced inflammatory cytokines such as interleukin-1 β (IL-1 β), tumor necrosis factor - α (TNF - α) and interleukin-6 (IL-6). Inhibited the activity of NF-kappaB.	(82)
	Salviae Miltiorrhizae Radix et Rhizoma (Salvia miltiorrhiza Polysaccharides)	Had anti-inflammatory effects	TNF-α, IL-6, iNOS, COX-2, NF-κB, p-p65, p-IκBa	Inhibited NF-κB signal pathway,the gene expressions and secretion of cytokines.	(83)
	Fritillariae Thunbergii Bulbus (Peimine)	Had anti-inflammatory effects	TNF-α, IL-6, IL-1β, IL-10, p38, ERK and c-jun N-terminal kinase (JNK) p65 and IκB	Blocked MAPKs and NF-κB signaling pathways.	(84)
	Paeoniae Radix Rubra (Paeoniflorin)	Had antiarthritis effects	Synoviocytes, IL-1, PGE2, IL-6, VEGF, GM-CSF,Gi, COX-2	Inhibited abnormal proliferation of synoviocytes and the production of Interleukin-1 (IL-1), prostaglandin E2 (PGE2), IL-6, vascular epidermal growth factor (VEGF) and GM-CSF by synoviocytes and reducing G protein (G i) and cyclo-oxygenase-2 (COX-2) expression.	(85)
	Platycodonis Radix (Platycodin D)	Enhanced the immunomodulatory activities of mouse lymphocytes and macrophages	Lymphocyte, macrophage, IL-2, IL-4, TNF-α	Promoted lymphocyte proliferation, enhanced phagocytosis of macrophage, and stimulated secretion of IL-2 and IL-4 in lymphocyte and secretion of TNF-α and IL-12 in macrophages.	(50)
	Saposhnikoviae Radix	Modulated immune functions		Increased nonspecific immunity and cell-mediated immunity and improved the spleen proliferation index.	(86)
	Glycyrrhizae Radix et Rhizoma (Glycyrrhizin)	May reduce the severity of an infection with COVID-19	ACE2	Reduced the expression of ACE2 in the lung.	(51, 52)
	Fructus Hordei Germinatus (glutamine-rich protein,hemicellulose-rich fiber)	Reduced the epithelial inflammatory response	STAT3, NFkB	Depressed signal transducer and activator of transcription 3 (STAT3) expression and inhibited nuclear factor kappa B(NFkB) binding activity.	(74)
	Crataegi Fructus (Maslinic acid)	Has anti-inflammatory properties	hGIIA-sPLA2-induced THP-1 cell	Inhibited hGIIA-sPLA2-induced THP-1 cell differentiation and migration.Binded and inhibited hGIIA-sPLA2 enzymatic activity.	(73)
	Massa Medicata Fermentata				
	Rhizoma Dioscoreae (Yam polysaccharides)	Has immunomodulatory effects	IL-2, TNF-α	Regulated the levels of IL-2 and TNF-α in tumor mice.	(87)

*LQG, Lianhua Qingwen Granules; HZDP, Huoxiang Zhengqi Dropping Pills; HXF, Hexin Formula; TZG, Tongzhi Granule; WM, Western medicine; NR, Not reported; TNF-α, tumor necrosis factor-α; IL-8, interleukin 8; ICAM-1, intercellular cell adhesion molecule-1; SFKs, Src family kinases; Th2 cell, T helper 2 cell; NF-κB signaling, nuclear factor-κB signaling; STAT3, signal transducer and activator of transcription 3; IRAK-4, interleukin-1 related acceptor kinase-4; IFN-β, interferon-β; ICAM-1, intercellular cell adhesion molecule-1; IL-6, interleukin-6; COX-2, cyclooxygenase-2; IL-1 β, interleukin-1 β; IL-1, Interleukin-1; PGE2, prostaglandin E2; VEGF, vascular epidermal growth factor; COX-2, cyclo-oxygenase-2*.

### Targeting the ACE2 and 3CLPro

HSYF is recommended as the first-line of treatment for COVID-19. Although due to time limitations, there have been only a few *in vivo* and *in vitro* experiments related to COVID-19, its efficacy in the clinical settings has been confirmed. Network pharmacology, a branch of pharmacology that uses network methods to analyze the synergistic relationship between drugs and diseases, and targets via “multi-component, multi-target, multi-pathway,” can help build a multi-dimensional network model of “drug–component–target–disease” to disclose the molecular mechanisms of multicomponent therapies, such as TCM (88, 89). HSYF was used to treat “cold–dampness stagnation in the lung” in COVID-19. Network enrichment analysis showed that HSYF components could to interleukin (IL)-6 and ACE2 (90). Since IL6 plays a central role in the acute inflammatory response, its potential inhibition could significantly improve prognosis in COVID-19 patients (91). SARS-CoV-2, binds to ACE2 in the human body through its expressed S-protein, facilitating its entry into host cells (92, 93). Blocking S-protein–binding to ACE2 may interfere with SARS-CoV-2 entry.

According to the network analysis, there are five ingredients (glycyrrhetinic acid, stigmasterol, indigo, β-sitosterol, and luteolin) in LQG that can act on ACE2. Molecular docking showed that these active ingredients could bind to ACE2, and their binding ability was higher than that of lopinavir, ritonavir, and ribavirin. 3CLpro is highly conserved in its genes and produces RNA-dependent RNA polymerase (RdRp) during the replication process of the coronavirus (94). Findings from gene network enrichment analysis showed that LQG could inhibit SARS-CoV 3C-like protease (3CLpro), thereby blocking the production of 16 non-structural proteins (95). Moreover, previous pharmacodynamics studies have demonstrated that LQG could significantly inhibit the activity of SARS-CoV *in vitro* culture and reduce the viral loads in the cytoplasm and cellular membrane (17).

**Figure 1:**
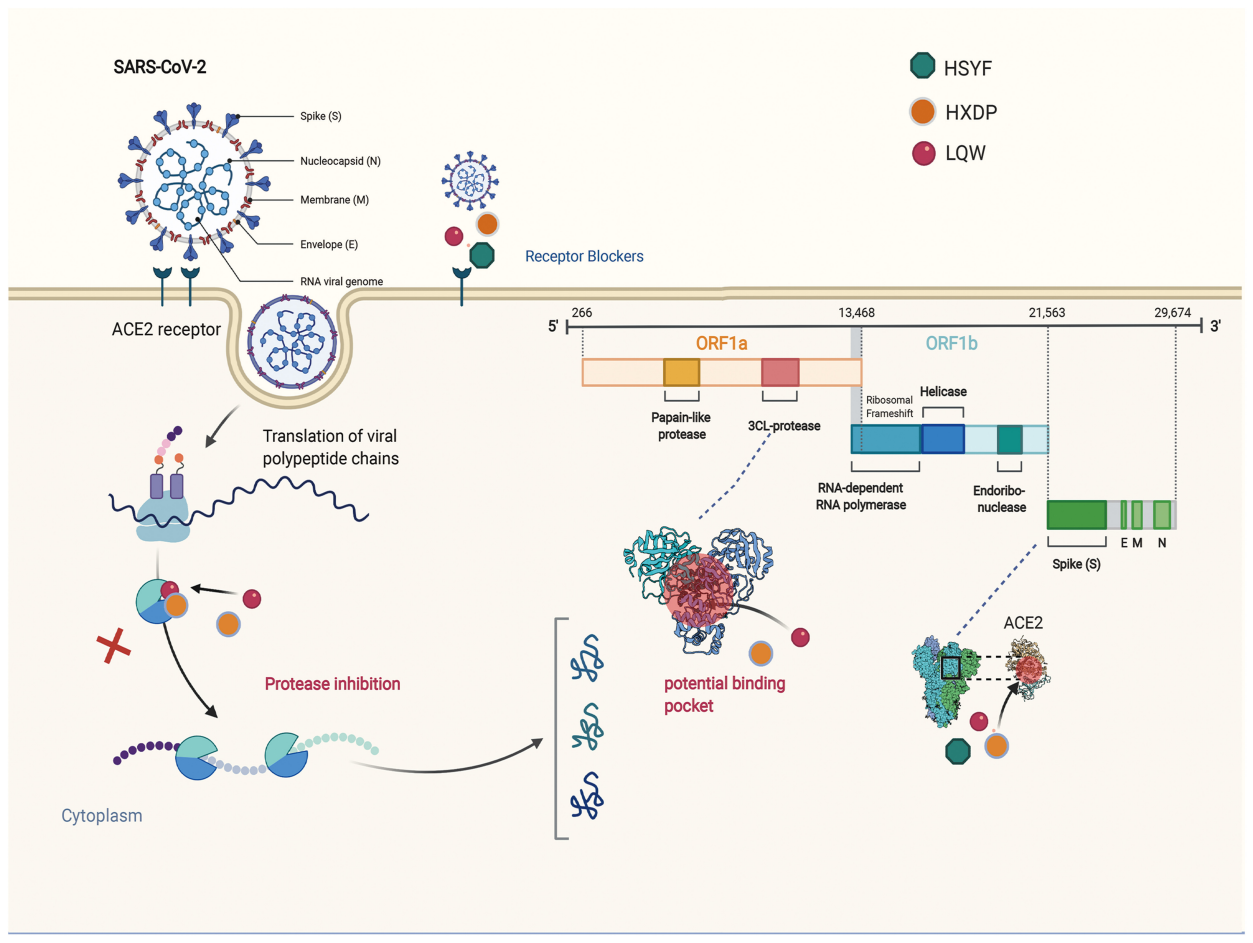
The schematic diagram illustrating proposed activity model of Herbal medicine in targeting 3C-like Proteinase (3CLPro) and Angiotensin converting enzyme two (ACE2). Green nodes represent Hanshiyi Formula (HSYF). Yellow nodes represent Huoxiang Zhengqi Dropping Pills (HZDP) and Lianhua Qingwen Granules (LQG).

Investigations of the effect of the HZDP on SARS-CoV-2 have yielded a consistent picture. Molecular docking showed that the five components of HXZQ (Elicorice glycoside E, naringenin, robinin, kaempferol, [(2R)-7-hydroxy-2- (4-hydroxyphenyl) chroman-4-one]), binds to 3CLpro, with an ability better than Remdesivir (96). These compounds contain flavonoid cores, and previous studies have demonstrated that flavonoids have a wide range of antibacterial and antiviral effects (97, 98). The schematic diagram illustrating proposed activity model of Herbal medicine in targeting 3C-like Proteinase (3CLPro) and Angiotensin converting enzyme two (ACE2) are presented in Figure 1.

### Targeting Cytokine

Cytokine storm syndrome (CSS) is a systemic inflammatory response that can be triggered by multiple factors such as infections and certain drugs (99). It is characterized by a sharp increase in the levels of pro-inflammatory cytokines, such as tumor necrosis factor (TNF)-α, IL-1, IL-6, IL-12, Interferon (IFN)-α, IFN-β, IFN-γ, monocyte chemoattractant protein-1 (MCP-1), and IL-8 (100). Cytokine storm syndromes are devastating clinical conditions that result from dysregulated immune responses to inflammatory and infectious triggers (101). These cytokines attack the immune system, causing acute respiratory distress syndrome and multiple organ failure (102). In the previous studies, cytokine storms have occurred during the infection process of SARS, Middle East Respiratory Syndrome and Ebola virus (103). Positive control of CSS is of great importance to the treatment, management, and prognosis of CSS.

A network pharmacology study showed that HSYF could play a role in immune regulation through proinflammatory and anti-inflammatory cytokines and exerts antiviral effects by regulating the hub targets IL6, TNF, IL10, mitogen activated protein kinase- (MAPK)-8, MAPK3, chemokine (CXCL)-8, caspase (CASP)-3, Prostaglandin-Endoperoxide Synthase (PTGS)-2, tumor protein p (TP)-53, and MAPK1 (90). IL6 and TNF play key roles in the cytokine storm. The most notable factor was IL6, which plays a key role in the cytokine storm, and is used as a clinical early warning index in the diagnosis and treatment of COVID-19. IL6 plays a central role in the acute inflammatory response, and a long duration of its release can also be used to assess the severity of infection and judge prognosis. Dynamic observation of IL-6 levels can assist in understanding the progression of infectious diseases and the response to treatment. In another network pharmacology, LQG could control inflammatory responses by regulating IL10, CD40 ligand, TNF, ACE2, IL-6, IFNA1, IL-2 and ACE (94, 95, 104). Moreover, LQG could block the activation of the MAPK signaling pathway, thereby inhibiting the release of inflammatory cytokines, and consequently reducing inflammation in tissues (105). Several studies have indicated that LQG can not only suppress the release of TNF-α, IL6, MCP-1 and CXCL-10 (106), but also reduce the expression of IL-1β, IL-2, IL-4 and IL-13 (107). Thus, suggesting that LQG can inhibit cytokine storm and relieve lung injury associated with inflammatory cell infiltration.

In addition, a retrospective cohort study showed that herbal medicine, (Ephedra sinica Stapf, Cinnamomum cassia Presl, Prunus armeniaca L. var. ansu Maxim., Poria, Pinellia ternata (Thunb.) Breit., Pogostemon cablin (Blanco) Benth., Eupatorium fortunei Turcz., Astragali Radix, Codonopsis Radix), could reduce the risk of morbidity in severe and critical COVID-19 (14). Although there is no direct evidence available for the effect of these herbal medicines on COVID-19, some indirect evidence supports the possibility of an association. In an *in vivo* experiment, Ephedra sinica Stapf effectively reduced the secretion of Th2 cytokines (IL-4, IL-5, IL-13) in mouse lung tissue and alveolar lavage fluid (108). In another *in vitro* anti-inflammatory experiment, Ephedra sinica Stapf inhibited the expression of IL-1β, IL-6, TNF-α, inducible nitric oxide synthase (iNOS), and macrophages (109). Gypsum fibrosum reduced the serum levels of TNF-α and IL-6, and IL-1β, TNF-α, and IL-6 in lung tissues in mice with systemic inflammatory response syndrome induced by lipopolysaccharide (110). Prunus armeniaca L. var. ansu Maxim. reduced the serum levels of TNF-α and soluble intercellular adhesion molecule-1 in rats with adjuvant arthritis, thereby slowing the development of tissue inflammation (111). The abovementioned evidence supports the role of these herbal medicines in targeting cytokines.

**Figure 2:**
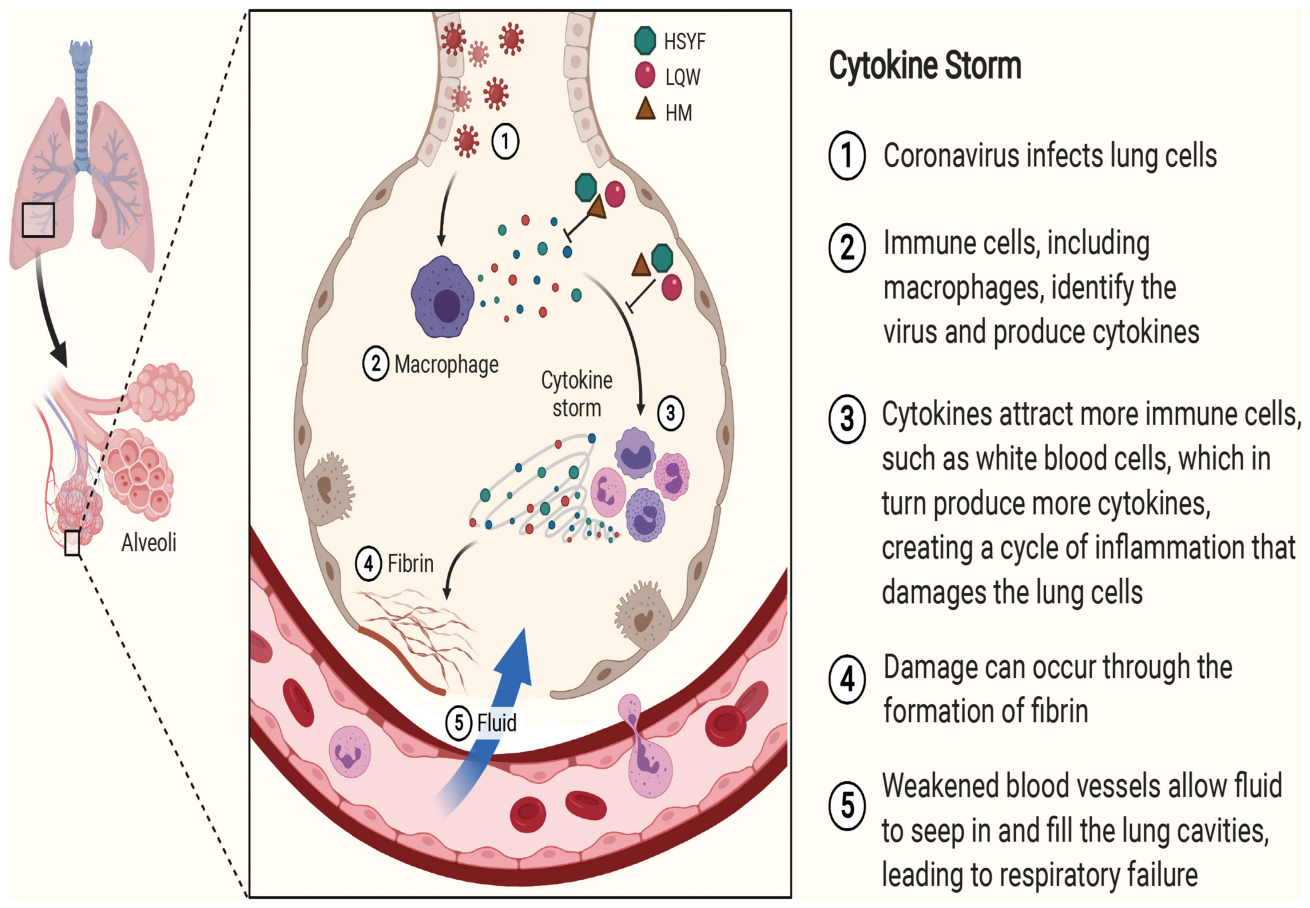
The schematic diagram illustrating proposed activity model of Herbal medicine in targeting cytokine. Green node represents Hanshiyi Formula (HSYF). Yellow node represents Huoxiang Zhengqi Dropping Pills (HZDP) and red node represents Lianhua Qingwen Granules (LQG).

The schematic diagram illustrating proposed activity model of Herbal medicine in targeting cytokine is presented in Figure 2.

### Targeting Acute Immune Responses to SARS-CoV-2

SARS-CoV-2 infects human lung epithelium through the receptor ACE2. The viral RNA activates endosomal and cytoplasmic sensors, Toll-like receptors (TLR)-3/7 and mitochondrial antiviral signaling protein, respectively. These receptors activate IFN regulatory factor (IRF) and Nuclear factor kappa B (NF-kB) to induce inflammatory cytokines, including interferon (IFN). Dendritic cell (DC) sample antigens migrate to lymphoid organs to trigger adaptive immunity. After recognizing antigens on DC or infected cells, CD8 T cells induce apoptosis (112–117). NF-κB plays a critical role in inflammation and the development of innate and adaptive immunity (118).

**Figure 3:**
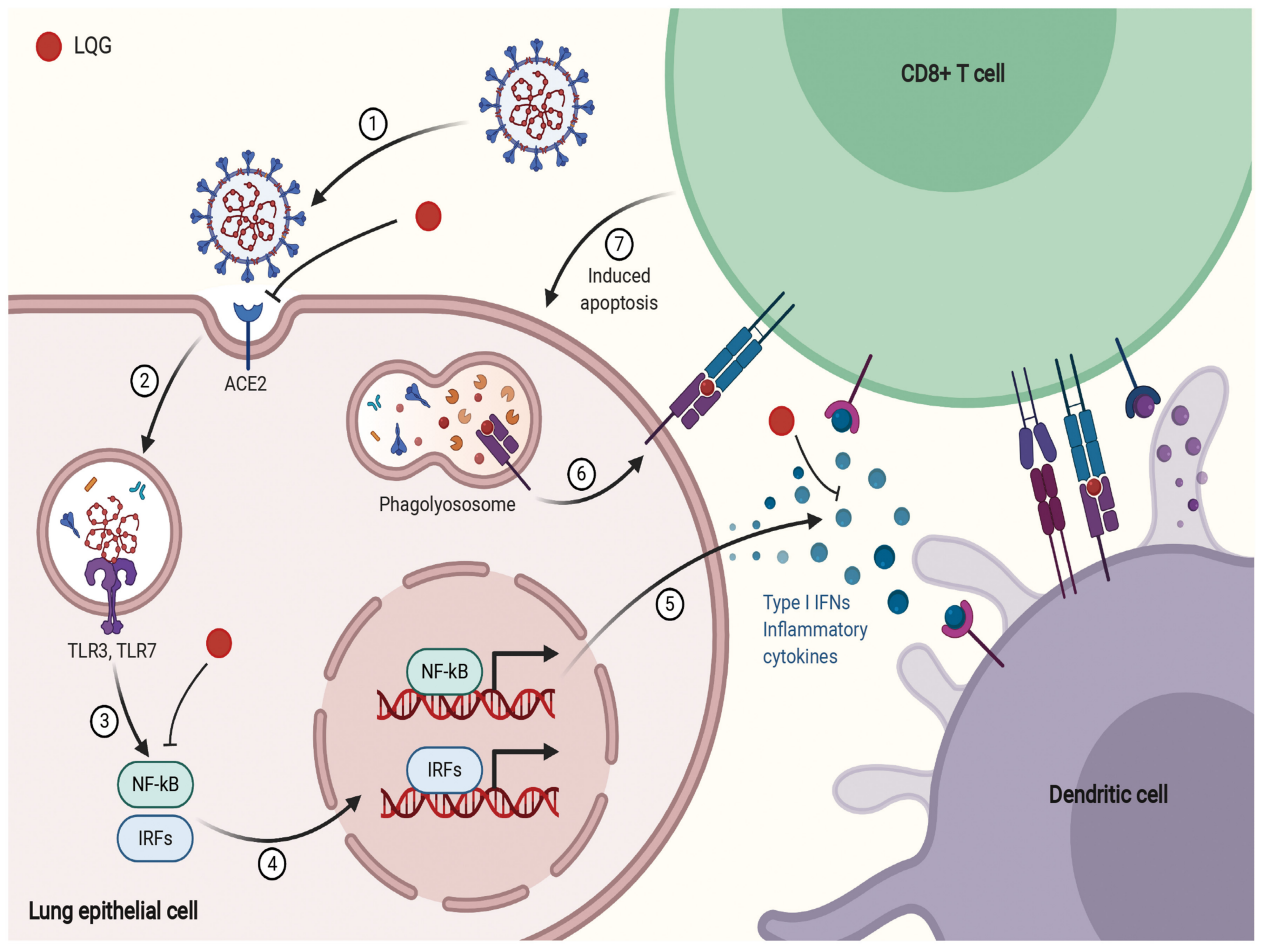
The schematic diagram illustrating proposed activity model of Herbal medicine in targeting acute immune responses to SARS-CoV-2. Red node represents Lianhua Qingwen Granules (LQG).

According to a network pharmacology study, LQG was involved in pathways related to innate immunity, including TLR, NF-κB, and type I interferon and such as Janus kinase/signal transducer and activator of transcription, MAPK1, CXCL2 (94, 95). Type I IFN is an early product of the innate immune response to viral infection (114). Activated NF-κB induces the expression of type I IFN, which triggers the migration of DC sample antigens to lymphoid organs (118). Forsythiaside, the active ingredient of LQG, can positively regulate the expression of interferon-α (IFN-α), hence exerting immune regulatory and antiviral effects (119). The schematic diagram illustrating proposed activity model of Herbal medicine in targeting acute immune responses to SARS-CoV-2 is shown in Figure 3.

### Targeting Pulmonary Fibrosis

**Figure 4:**
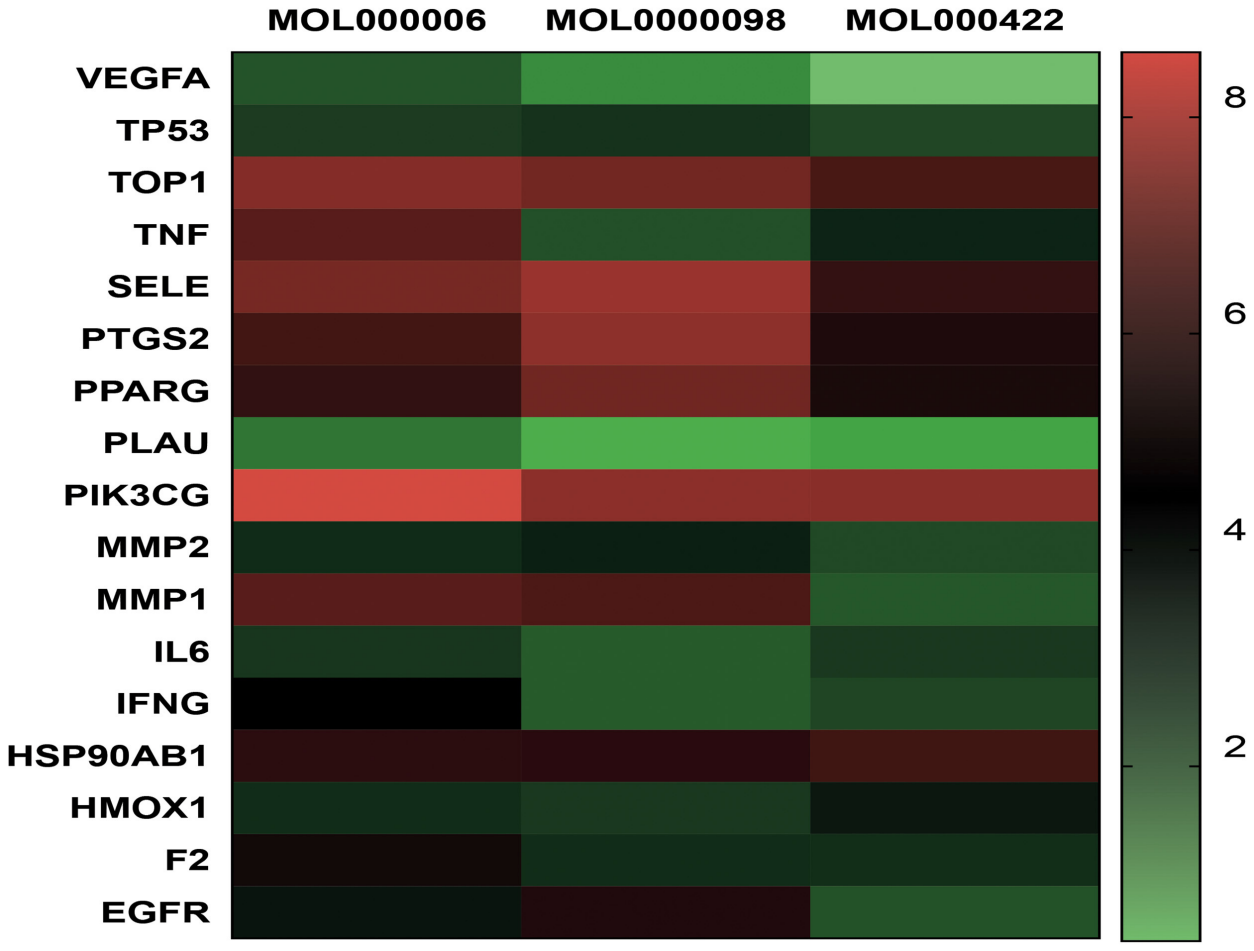
Heat map of Molecular docking. Molecular models of the binding of quercetin (MOL0000098), kaempferol (MOL000422), luteolin (MOL000006) with TOP, MMP2, MMP9, IFNG, SELE, PLAU, VEGFA, HMOX1, F2, TNF, TP53, PPARG, PIK3CG, IL6, PTGS2, HSP90AB1, and EGFR, respectively.

**Figure 5:**
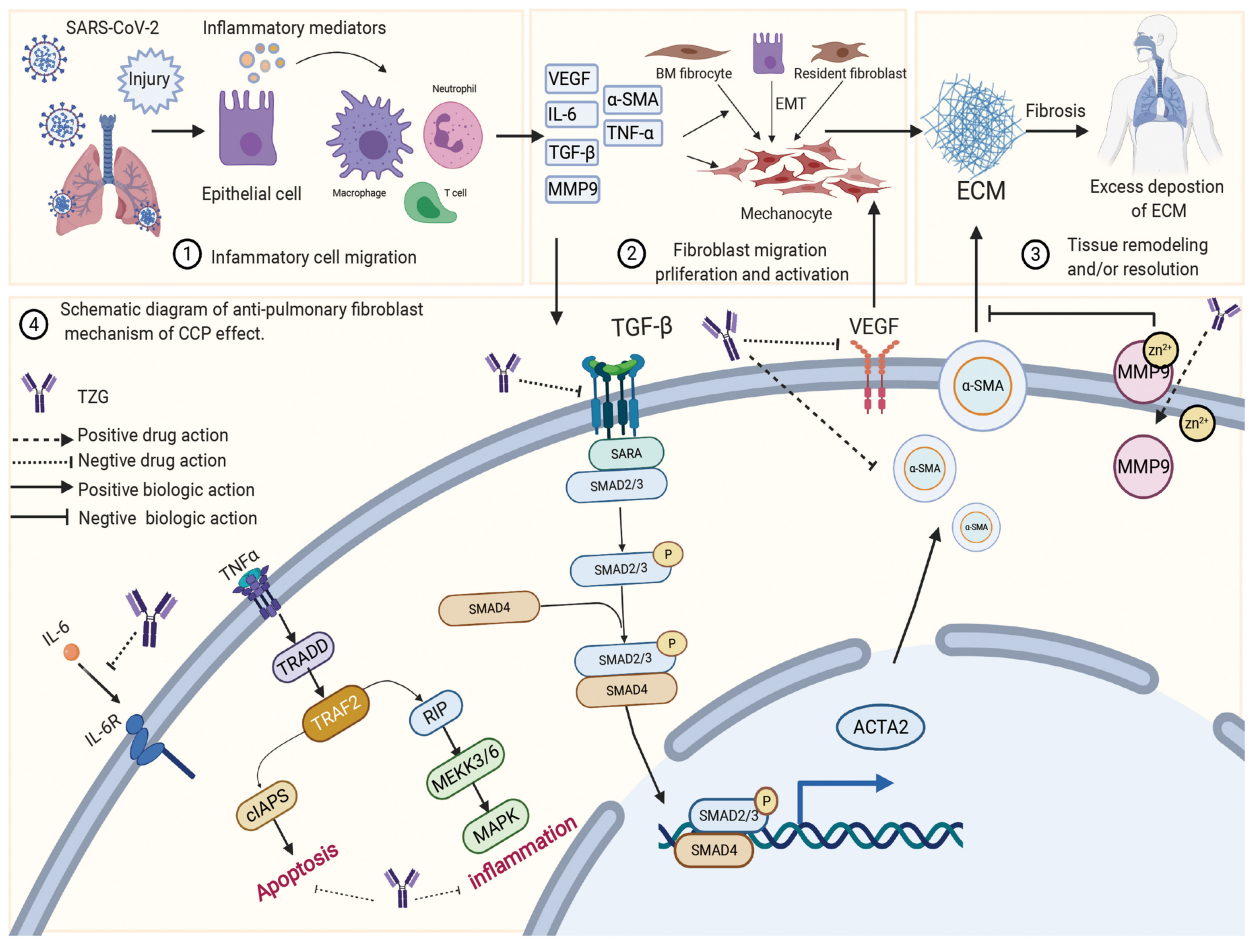
The schematic diagram illustrating proposed activity model of Tongzhi Granule (TZG) in pulmonary fibrosis.

TZG was widely used for patients with COVID-19 who were in the recovery period and were at high risk of pulmonary fibrosis (PF). Using a network pharmacology analysis, we investigated the anti-pulmonary fibrosis mechanisms of TZG. Findings indicated that TZG could inhibit the expression of vascular endothelial growth factor (VEGF), TNF-α, IL-6, MMP9, and TGF-β1 via the VEGF, Toll-like receptor, MAPK, and TGF-β1 signaling pathways. The binding ability and of herbal components to core protein targets was validated by molecular simulations. On molecular docking using Surflex-Dock modeling, a docking score of >3 signified a stable compound with strongbinding. Quercetin, kaempferol, and luteolin exhibited high binding activity to targets associated with PF. For example IL-6 (score = 3.0236, 3.6316, 3.7055, respectively), TNF-α (score = 3.2116, 3.9889, 5.9409, respectively), VEGF (score = 3.0175, 3.844, 3.1564, respectively), and MMP9 (score = 5.7384, 3.079, 5.9618, respectively). Detailed blinding scores were shown in the Heat map in Figure 4, and the potential anti-pulmonary fibrosis mechanism of TZG is summarized in Figure 5.

## Discussion

In the battle of the epidemic in Wuhan, the clinical experience of TCM in COVID-19 patients can be used as a valuable reference. Tong proposed that the COVID-19 epidemic in Wuhan demonstrated cold-damp characteristics in terms of disease, environment, and the human body. The theory was proposed to interpret the characteristics of the epidemic from a TCM perspective, and to develop the treatment accordingly. Under the guidance of cold-damp plague theory, a series of TCM prescriptions, for varying stages, such as the mild, moderate, severe, critical, and recovery, were developed. TCM drugs, namely HZDP, LQG, HSYF, HXF, and TZG have been utilized in the treatment of COVID-19 patients (12–14). For suspected and diagnosed cases of COVID-19, HZDP+LQG was significantly more efficacious, when compared to LQG alone and western medicine; besides, it reduced the proportion of patients who progressed to severe. Although LQG and western medicine could alleviate cold symptoms, the HZDP+LQG group had a unique advantage in improving damp symptoms such as nausea, vomiting, and limb soreness. The results indicated the HZDP+LQG was effective in dispelling cold and removing dampness in patients with COVID-19. For mild and moderate cases of COVID-19, HSYF was developed; it eliminated cold-dampness from the body and relieved lung inflammation. We founded that it significantly reduced the progression in mild and moderate COVID-19 cases to develop severe conditions, which indicated that HSYF may have positive effects in the treatment of the epidemic. For severe and critical patients, HXF was developed to dispel cold-dampness and invigorate healthy *qi*. We found that, with the use of HXF, the mortality in severe and critical cases could be reduced by 82.2% as estimated in a retrospective observational cohort study. For recurrent RT-PCR positive cases, TZG was developed to supplement healthy qi and expel residual cold-damp pathogens from the body. TZG significantly reduced the RT-PCR test results to 2.8% in patients recovered from COVID-19.

Despite the fact that there is enough clinical evidences on the utility of TCM in COVID-19, the underlying mechanisms, from the perspective of modern science, are yet to be elucidated. Network pharmacology has provided a feasible reference. Network pharmacology revealed that HZDP can inhibit the replication of SARS-CoV-2 by interfering with the ACE2 enzyme and 3CL hydrolase (95). 3CLpro is a cysteine protease, a functional protein that mediates the hydrolysis of replicase polypeptides 1a and 1ab and during virus replication and proliferation (95). 3CLpro is highly conserved in its genes and produces RNA-dependent RNA polymerase (RdRp) during the replication of coronavirus. Therefore, 3CLpro can serve as a target for the drug design, and provide a breakthrough in the development of anti-SARS-COV-2 drugs (37, 38, 91, 97). According to the results of molecular docking, five components of HZDP could bind with SARS-COV-2 3CLpro; the binding ability was better than the control drug Remdesivir. LQD could exert anti-inflammatory activity to treat COVID-19, mainly through reducing the levels of inflammatory response factors IL-8, IL-17, IL-23 and TNF-α, lowering the levels of IL-8 and IL-17 in the blood, and inhibiting virus-induced activation of NF-kB and gene expression of IL-6, IL-8, TNF-a, and IP-10, which can reduce the inflammatory response and slow down the damage of inflammatory response exudate to lung function (94, 96, 104).

The arachidonic acid metabolic pathway mediates the production of a variety of inflammatory response factors (120–122). Inhibiting this may decrease inflammation in patients with COVID-19. For mild and moderate COVID-19 cases, HSYF successfully reduced the progression to severe status, and alleviated symptoms in patients by exerting anti-viral effect, immune regulation and anti-inflammatory pathways (90). A correlation between blood glucose control and prognosis in patients with co-existing COVID-19 and diabetes has been reported (123); HSYF could target the AGE-RAGE signaling pathway in such patients. Molecular docking indicated that quercetin and luteolin, and L-tyrosine and L-phenylalanine had good binding activities to IL6 and ACE2, respectively. IL-6 levels can assist in understanding the progression of infectious diseases and the response to treatment (124). All these potential mechanisms may be related to the therapeutic effect of HSYF.

Notably, in patients who have experienced and survived a COVID-19, PF has been observed, which may progress to chronic and severe interstitial lung disease. A meta-analysis showed that there is an obvious association between the development of PF and respiratory viral infections (125). It is well-known that SARS-CoV-2 invades host cells and interacts with ACE2; ACE2 is highly expressed in type II lung cells and directly participates in the occurrence and development of inflammation and PF (126, 127). Preventing the occurrence of PF in patients recovering from SARS-CoV-2 infection is of vital importance. TZG is formulated for fibrosis in COVID-19 patients at the recovery stage.

The pathological process of PF can be roughly divided into three stages. The first is the diffuse damage of vascular endothelial cells and alveolar epithelial cells by pathogenic factors, which initiates the inflammatory immune response. Second, a variety of inflammatory cells release various cytokines and inflammatory mediators, expanding tissue damage and causing interstitial hyperplasia. The third is the migration and proliferation of fibroblasts and endothelial cells, and the metabolic disorders of collagen and other extracellular matrices, which aggravate inflammatory damage and proliferation in a feedback manner. Eventually, the process could lead to the replacement and reconstruction of normal lung tissue. These processes involved in these stages exist simultaneously (128), and their interaction generates other mediators involved in the inflammatory response, such as TNF-α and IL-6, which directly or indirectly promote the synthesis of ECM through interaction with other cytokines (129). More importantly, several studies have robustly documented that silencing the expression of TGF-β1 reduces inflammation and slows the progression of PF (130). Network pharmacology suggested that TZG can reduce the expression of TGF-β1, α-smooth muscle actin (α-SMA) and TNF-α, and inhibit alveolar cell apoptosis, and hence reduce lung inflammation and fibrosis damage.

In this review, we explained COVID-19 in Wuhan, according to the cold-dampness theory of TCM, and offered a series of clinical evidence to support our opinion. Furthermore, the underlying mechanisms, in the light of modern pharmacology, were discussed to support the utility and efficacy of TCM. However, there are limitations. First, since COVID-19 is a global pandemic that has been widely spread in countries with different climates, the cold-damp nature of the epidemic in Wuhan cannot represent the characteristics of the disease in other regions of the world. One point of view to explain the epidemic transmission of people in hot and humid areas of the globe is that increased humidity in the atmosphere could reduce the air temperature, thus indirectly influencing disease susceptibility (25). Despite meteorological, environmental and etiological factors related to the disease, physical defense measures such as face masks, social distancing, and contact tracing are also important factors affecting the transmission and progression of the epidemic (131). Thus, in this review, we adopted cluster study approaches by enrolling COVID-19 patients in the Wuhan area, to provide objective clinical evidence for TCM treatments, guided by the theory of cold-dampness plague. Second, although the clinical efficacy of these TCM prescriptions guided by cold-damp plague theory has been confirmed clinically as well as network pharmacology, there is still a lack of high-level evidence to evaluate the effectiveness and safety of the TCMs. The mechanisms of these drugs have been elucidated based on network pharmacology and molecular docking speculations, which have not yet been experimentally verified. In response to the above problems, the effectiveness and safety of TCM in the treatment of COVID-19 needs to be further evaluated in a large-scale RCT. Further, the anti-coronavirus mechanisms should be further verified through cell experiments, animal experiments, and multi-omics studies, which can provide the basis for new drug development of COVID-19, and also provide a new option for the prevention and control of the epidemic.

## Author Contributions

YZhe and DJ wrote the draft of the manuscript and contributed equally to this work. FL and XT designed the study and as the corresponding authors. JL, YZha, and JT participated in the revision of the manuscript. All authors contributed to the article and approved the submitted version.

## Conflict of Interest

The authors declare that the research was conducted in the absence of any commercial or financial relationships that could be construed as a potential conflict of interest.
